# Transmissibility of Livestock-associated Methicillin-Resistant *Staphylococcus aureus*

**DOI:** 10.3201/eid1911.121085

**Published:** 2013-11

**Authors:** David J. Hetem, Martin C.J. Bootsma, Annet Troelstra, Marc J.M. Bonten

**Affiliations:** University Medical Center Utrecht, Utrecht, the Netherlands (D.J. Hetem, M.C.J. Bootsma, A. Troelstra, M.J.M. Bonten);; University of Utrecht, Utrecht (M.C.J. Bootsma)

**Keywords:** livestock, MRSA, community, bacteria, methicillin-resistant Staphylococcus aureus, transmission

## Abstract

Previous findings have suggested that the nosocomial transmission capacity of livestock-associated methicillin-resistant *Staphylococcus aureus* (LA-MRSA) is lower than that of other MRSA genotypes. We therefore performed a 6-month (June 1–November 30, 2011) nationwide study to quantify the single-admission reproduction number, *R_A_,* for LA-MRSA in 62 hospitals in the Netherlands and to compare this transmission capacity to previous estimates. We used *spa* typing for genotyping. Quantification of *R_A_* was based on a mathematical model incorporating outbreak sizes, detection rates, and length of hospital stay. There were 141 index cases, 40 (28%) of which were LA-MRSA. Contact screening of 2,101 patients and 7,260 health care workers identified 18 outbreaks (2 LA-MRSA) and 47 secondary cases (3 LA-MRSA). *R_A_* values indicated that transmissibility of LA-MRSA is 4.4 times lower than that of other MRSA (not associated with livestock).

Methicillin-resistant *Staphylococcus aureus* (MRSA) is one of the leading causes of nosocomial infections and leads to considerable illness, death, and health care costs (*1**,**2*). The worldwide epidemiology of MRSA has changed as MRSA originating in the community has increased. These community-associated MRSA (CA-MRSA) strains are replacing their hospital-associated counterparts in hospitals in the United States; the major dominant clone is MRSA strain USA300 (*3*). In recent years, another MRSA clone, which originated in the community and is associated with exposure to livestock, has emerged in different countries worldwide, including the United States (*4**,**5*). Even more worrying, countries with a historically low prevalence of MRSA, like the Netherlands and Denmark, have seen an increase in livestock-associated MRSA (LA-MRSA), belonging to clonal complex 398 (*5*). In the Netherlands, LA-MRSA accounted for 39% of all new MRSA isolated in 2011 (*6*). Yet almost all isolates have been detected through screening, and in 2009, nine infections were caused by MRSA sequence type 398 (*7*). Invasive infections caused by LA-MRSA include endocarditis, osteomyelitis, and ventilator-associated pneumonia (*8**,**9*). 

It has been suggested that in the Netherlands, this MRSA genotype has a lower capacity than other genotypes for nosocomial transmission (*10**,**11*). The lower transmission rates might result from differences in human host characteristics or from a lack of pathogen adaptation to the human host, which could change over time (*12*). In a previous study in the Netherlands in 2005, we quantified the transmission capacity, expressed as the single-admission reproduction number per hospital admission, *R_A_*, and obtained values of 0.16 for LA-MRSA and 0.68 and 0.98 for MRSA not associated with livestock (hereafter referred to as other MRSA) (*10*). We therefore performed a nationwide study to quantify *R_A_* for LA-MRSA in hospitals in the Netherlands and to compare this transmission capacity to our previous estimates.

## Methods

### Data Collection

Medical microbiologists and infection control practitioners in all 91 hospitals in the Netherlands were contacted and asked to collect data concerning MRSA outbreaks and the results of subsequent contact screening retrospectively during June–August 2011 and prospectively during September–November 2011. A standardized Web page was used for data collection. An index case-patient was defined as a hospitalized patient colonized or infected with MRSA and treated without use of barrier precautions. Age, sex, and number of days hospitalized from MRSA detection through discharge were obtained. According to the guidelines in the Netherlands, identification of a MRSA index case-patient initiates contact screening among contact patients and health care workers (HCWs) (*13*). The numbers of screened patients and HCWs and the number of secondarily colonized patients and HCWs were obtained. A secondary case-patient was defined as a patient with MRSA with a *spa* type identical or related to that from the index case-patient, detected during contact screening of a patient or HCW. Newly identified MRSA carriers with MRSA *spa* types that were unrelated to that of an index case-patient were considered incidental findings. The study was approved by the medical research ethics committee of the University Medical Center Utrecht.

### MRSA Genotyping

For all MRSA isolates, single-locus DNA sequencing of the repeat region of *Staphylococcus* protein A gene (*spa* typing) was performed by the national reference laboratory of the Netherlands (National Institute for Public Health and the Environment [RIVM]), as described (*14*), by use of the Ridom StaphType program (www.ridom.de) to allocate *spa* types. MRSA isolates were considered to be associated with livestock if they had a livestock-associated *spa* type: *t011, t034, t108, t567, t571, t588, t753, t753, t779, t898, t899, t943, t1184, t1197, t1254, t1255, t1451, t1456, t1457, t2123, t2287, t2329, t2330, t2383, t2582, t2748, t2971, t2974, t3013, t3014, t3053, t3146*, or *t3208* (*15**–**17*). All other *spa* types were considered to not be associated with livestock. To identify potentially unknown livestock *spa* types, we used Bionumerics 5.1 (Applied-Maths, Sint Maartens-Latem, Belgium) to create a *spa*-based minimal spanning tree  of *spa* types considered livestock-associated and the *spa* types of index cases (Technical Appendix Figure). Genes encoding for Panton-Valentine leukocidin (PVL), *LukS-PV*, and *LukF-PV* were identified by the reference laboratory, as described (*18*).

### Model

To estimate the strain-specific transmission capacity *R_A_* value, we used a previously described mathematical model based on queueing theory (*19*). *R_A_* is defined as the average number of secondary cases caused by 1 primary case (the index case) when other patients are susceptible during a single hospital admission of the primary case-patient (*20*). In this model, 3 rates determine the spread of MRSA in the hospital setting: the rate at which the MRSA strain spreads, the rate at which MRSA colonization of a patient is detected (e.g., microbiological cultures), and the rate at which a colonized patient can no longer be detected. The model predicts that the distribution of the number of patients colonized at the time of detection of the index case is geometrically distributed. The parameter of the geometric distribution of detected outbreak sizes was determined by using maximum-likelihood estimations. Small detected outbreak sizes could correspond to either low transmission potential or high detection rate.

Patients with MRSA remain colonized during their hospital stay; therefore, the infectious period ends at the time of discharge. Genotype-specific discharge rates were calculated from admission and discharge data for index case-patients admitted to participating hospitals during the study period. The detection rate was based on all blood, respiratory tract, and wound cultures conducted during 2011 at the University Medical Center Utrecht. The upper detection limit consists of all these cultures divided by the total number of patient days in 2011. By combining the detection and discharge rate with the parameter of geometric distribution, we could calculate *R_A_*. Details about the model are included in the online Technical Appendix. 

### Statistical Analyses

Categorical variables were assessed 2-sided by using χ^2^ or Fisher exact tests, as appropriate; a cutoff value of p< 0.05 was applied for significance. Continuous variables were analyzed by using the Mann-Whitney U test. Confidence intervals were calculated by using the profile-likelihood method. To test whether our assumption of a geometrical distribution of the detected outbreak sizes is justified by the data, we performed the Anderson-Darling goodness-of-fit test. Data were analyzed by using SPSS for Windows version 20.0 (IBM Corp., Armonk, NY, USA). Further details about the statistical methods used are included in the online Technical Appendix.

## Results

A total of 62 (69%) of the 91 hospitals in the Netherlands participated in the study, yielding data for 372 months of MRSA policy. During the 6-month study period, 158 MRSA index case-patients were identified in 57 hospitals, and none were identified in the other 5 hospitals. These numbers imply that, on average, in each hospital an index case was detected every 2.5 months. Two index case-patients were excluded because subsequent contact screening was not performed, and 15 index case-patients were excluded because barrier precautions were implemented on the day of admission. For these 15 index case-patients, contact screenings of 55 patients and 293 HCWs had identified 1 MRSA-colonized HCW with an unrelated MRSA genotype. For the remaining 141 index case-patients, 9,361 contacts (2,101 patients and 7,260 HCWs) were screened.

In total, 65 *spa* types were identified among the 141 index cases; the most common were t011 (n = 25 [18%]), t008 (n = 12 [9%]), and t002 (n = 7 [5%]). A total of 40 (29%) isolates had *spa* types indicative of LA-MRSA; the most prevalent were t011 (n = 25), t034 (n = 6), and t108 (n = 6) (Table 1).

**Table 1:** Genotypes of methicillin-resistant *Staphylococcus aureus* from index and secondary case-patients, the Netherlands*

*spa-*type	No. (%) with PVL	No. index case-patients, n = 141	No. outbreaks, n = 18	No. (%) secondary cases, n = 47
LA-MRSA				
t011	0/24	25	1	2 (4)
t034	0/5	6	0	0
t108	0/5	6	1	1 (2)
t899	0/2	2	0	0
t2330	0/1	1	0	0
Other MRSA				
t008	8/12 (67)	12	0	0
t002	1/7 (14)	7	2	4 (2)
t032	0/5	5	1	6 (13)
t064	0/5	5	1	5 (11)
t1081	0/3	5	3	14 (31)
t688	0/3	4	0	0
t038	1/3 (33)	3	1	1 (2)
t267	0/3	3	0	0
t001	0/1	2	0	0
t018	0/2	2	0	0
t179	0/2	2	1	1 (2)
t447	0/2	2	1	1 (2)
t1430	0/2	2	0	0
t1469	0/2	2	0	0
Singletons	14/42 (33)†	45	6‡	12 (24)

*PVL, Panton-Valentine leukocidin; LA-MRSA: livestock-associated methicillin-resistant *Staphylococcus aureus*. †PVL positive: t022, t040, t044, t054, t131, t311, t318, t437, t657, t690, t791, t852, t2815, t3523, t7277;  *‡Spa* types causing outbreaks: t003, t088, t311, t1399, t7277, t9634.

*Luk*-PV genes, indicative of PVL, were detected in 24 (18%) of 131 isolates investigated, all categorized as not being LA-MRSA strains. Among 12 MRSA *spa* type t008 isolates, PVL positivity was detected in 8 (67%) (Table 1), and among 10 (7%) MRSA isolates, the presence of PVL was undetermined.

Mean age among all index case-patients was 53 years. Among patients with LA-MRSA and other MRSA genotypes, no significant differences were found except for sex (Table 2). Among index case-patients with LA-MRSA genotypes, 83% were male, compared with 56% case-patients with other MRSA (p = 0.004). No statistically significant differences were found in length of hospital stay (p = 0.222) and number of days in hospital without barrier precautions (p = 0.503) between index case-patients with LA-MRSA and patients with other MRSA genotypes (Table 2).

**Table 2:** Characteristics of index case-patients with LA-MRSA and other MRSA genotypes*

Characteristic	LA-MRSA, n = 40	Other MRSA, n = 101	p value
*R_A_* (95% CI)	0.12 (0.03–0.30)	0.52 (0.38–0.69)	NA
Age, y	56	52	0.337
Male, no. (%)	33 (83)	57 (56)	0.004
Length of stay (median), d	13	10	0.222
Days not in isolation (median)	5	6	0.503

*LA-MRSA: livestock-associated methicillin resistant *Staphylococcus aureus*; *R_A,_* single-admission reproduction number; NA, not applicable.

Among 141 postexposure screenings, MRSA carriers were identified for 18 (13%) case-patients, yielding 39 newly identified colonized patients and 34 newly identified colonized HCWs with MRSA. Screening of index case-patients with LA-MRSA identified 15 (21%) carriers, and screening of index case-patients with other MRSA identified 58 (79%) carriers. Of these 73 MRSA carriers, 47 (64%) were colonized with a MRSA *spa* type that was identical to that of the corresponding index case-patient; 3 patients had *spa* types matching those of 2 index case-patients with LA-MRSA, and 44 had *spa* types matching those of 16 index case-patients with other MRSA. Transmission of MRSA (i.e., outbreaks) was documented for 18 index patients; the largest outbreak consisted of 12 secondary cases (8 patients and 4 HCWs, *spa* type t1081), and most outbreaks (11 [61%] of 18) consisted of only 1 secondary case (Figure). Contact screening for 1 index case-patient with LA-MRSA (t011) revealed 1 outbreak consisting of 3 patients with a MRSA genotype (t067) that was not LA-MRSA. These newly identified cases of MRSA carriage were considered to be not associated with the index case with an LA-MRSA genotype.

**Figure:**
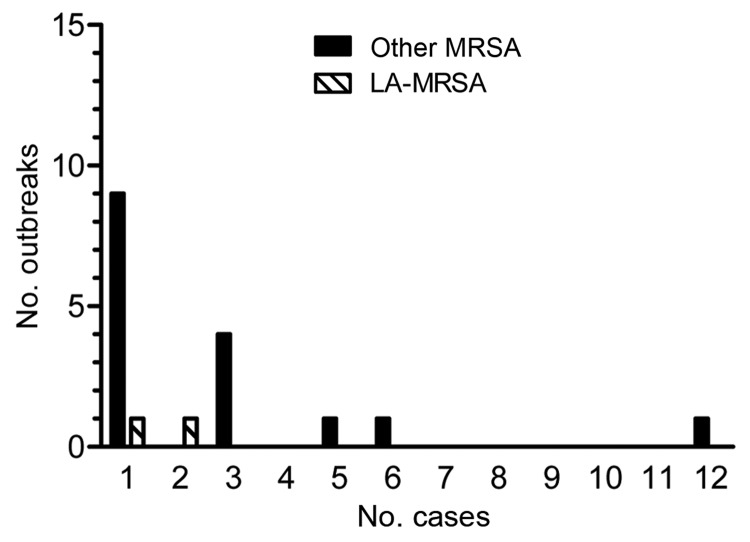
Number of outbreaks and outbreak sizes (number of cases, excluding the index case). LA-MRSA, livestock-associated methicillin-resistant *Staphylococcus aureus*; other MRSA, MRSA not associated with livestock.

During 2011, a total of 6,819 blood, 4,828 respiratory tract, and 1,132 wound cultures were performed. For the upper limit of detection, we used only 1 culture per patient per day, yielding 11,903 relevant cultures, divided by the number of patient-days (241,319) (Technical Appendix Table A).

The ratio between detection and discharge rates did not differ much between patients with LA-MRSA and other MRSA (Technical Appendix Table A). The parameter for geometric distribution for LA-MRSA and other MRSA is also provided in the online Technical Appendix. There was no reason to reject the hypothesis of a geometrically distributed outbreak size for LA-MRSA; but the hypothesis was rejected for other MRSA (p<0.05).

Based on the genotype-specific ratio between detection and discharge rates, *R_A_* values were 0.43 (95% CI 0.32–0.56), 0.12 (95% CI 0.03–0.31), and 0.52 (95% CI 0.38–0.69) for all 27 genotypes, LA-MRSA, and other MRSA, respectively. According to these *R_A_*-values, the transmissibility of LA-MRSA was considered 4.4 times lower than that of other MRSA (0.12/0.52). The *R_A_* value for PVL-positive strains was 0.31 (95% CI 0.14–0.58).

## Discussion

Using data from 62 hospitals in the Netherlands, comprising 372 months of MRSA policy, we determined that livestock-associated MRSA genotypes, compared with other MRSA genotypes, are 4.4 times less likely to spread in the hospital. Our findings in this study add substantial knowledge to findings from our previous study of hospitals in the Netherlands in 2005 (*10**,**11*). The current study included a larger cohort of hospitals and genotyping of all isolates. In our previous study, we compared *sma*I non-typeable MRSA to other MRSA genotypes without further genotyping. The genotyping demonstrates the heterogeneity in index cases with MRSA not associated with livestock. Moreover, in the current study, we collected more detailed patient information, such as admission and discharge dates and the number of days that index and secondary case-patients were treated without barrier precautions, which enabled more precise estimation of parameters. Absence of significant differences in age, length of hospital stay, or number of days not spent in isolation between index case-patients with LA-MRSA and those with other MRSA reduces the possibility that the differences in transmission capacity resulted from differences in patient characteristics. The only difference was that LA-MRSA index case-patients were more likely to be male, reflecting sex distributions among pig farmers and veal calf farmers.

For this study, we made several assumptions. First, no differentiation was made between patients and HCWs. Both are at risk for colonization with MRSA; however, infectious period and infectivity may differ. Second, all carriers were assumed to be equally infectious; whereas, superspreaders could play a major role in the transmission of MRSA in certain outbreaks. The consequences of these assumptions have been discussed in detail elsewhere (*10*). 

This study has several limitations. For this model to work, MRSA outbreaks must be rare and rigorous screening must be performed after the identification of an index case. If multiple outbreaks of the same genotype occur on the same ward, *R_A_* would be an overestimation. Here, s*pa* typing was used to identify cases of transmission between index and secondary case-patients. Among LA-MRSA, 63% were *spa* type t011; whereas, other MRSA consist of many different *spa* types. The high prevalence of LA-MRSA in pig-dense areas combined with the homogeneity of *spa* types could lead to an actual overestimation of these transmission events (and the estimated *R_A_* values of LA-MRSA).

LA-MRSA comprise a well-defined set of *spa* types, most commonly t011, t034, and t108; whereas, other MRSA comprise a highly heterogeneous group with hospital-associated genotypes and PVL-positive, community-associated genotypes (*21*). Almost 25% of all other MRSA were PVL positive, which is considered a characteristic of community-associated MRSA. Although 25% seems high, the actual incidence of index case-patients with PVL-positive MRSA was 24 in 379 hospital months, comprising an average of 1 index case per 16 months per hospital. In contrast to LA-MRSA and hospital-associated MRSA, there are no established risk factors in the Netherlands for colonization with CA-MRSA, and unknown carriers of these genotypes will not be screened when admitted to hospital (*13*). Although another study from the Netherlands reported a high number of PVL-positive isolates in MRSA-colonized patients without risk factors as described in the national guidelines (*13**,**22*), our findings demonstrate that PVL-positive strains do not constitute a major risk for health care settings in the Netherlands because the introduction rate and the *R_A_* in the absence of barrier precautions (*R_A_* for PVL-positive strains 0.31, 95% CI 0.14–0.58) are low. Nevertheless, if admission rates increase, outbreaks could emerge despite *R_A_* values <1 (*20*).

*spa* type t1081 was associated with the highest number of outbreaks and with most secondary cases. This *spa* type has also been associated with outbreaks in nursing homes across the Netherlands. For s*pa* type t1081, the MIC for cefoxitin (data not shown) is low (3 mg/L [range 3–8 mg/L]), hampering laboratory detection during routine procedures, which might have contributed to the high number of secondary cases found with this *spa* type.

Whole-genome analyses of multiple sequence type 398 *S. aureus* strains suggests that LA-MRSA originated from methicillin-susceptible *S. aureus* that crossed species barriers from humans to livestock, where it acquired resistance traits (*23*). It has been hypothesized that the transition from humans to animals was associated with the loss of several human immune evasion genes, carried on phage φSa3, which may prevent human niche adaptation of LA-MRSA (*24*). Whether this loss is associated with the lower *R_A_* remains to be determined.

The epidemiology of CA-MRSA in Europe differs markedly from that in the United States; >50% of community-acquired *S. aureus* infections in Europe are caused by a few PVL-positive clones (*25*). There is a paucity of data on the nosocomial transmission capacity of CA-MRSA. In hospitals in the Netherlands, though, the estimated *R_A_* of CA-MRSA, consisting of a heterogeneous group of genotypes, was estimated to be 0.07 (95% CI 0.00–0.28) (*26*), and in the hospitals participating in the present study, the *R_A_* value of PVL MRSA strains was 0.31, 95% CI 0.14–0.58. The differences between Europe and the United States regarding the epidemiology of PVL-positive CA-MRSA, therefore, remain unexplained.

Current guidelines in the Netherlands recommend MRSA screening for all patients with professional exposure to livestock, and many hospitals treat such patients in isolation while screening results are pending (i.e., preemptive isolation). In a previous multicenter study in the Netherlands, we demonstrated the cost-effectiveness and safety of not preemptively isolating patients when using rapid diagnostic testing (*27*). That evaluation included all MRSA genotypes: LA-MRSA and other MRSA. The confirmation of the lower transmissibility of LA-MRSA (in combination with the low *R_A_* value) and the results of the previous study provide evidence that preemptive isolation may not be necessary for LA-MRSA, which would substantially enhance the feasibility of this highly successful infection control policy.

Technical Appendix Statistical methods used to calculate single-admission reproduction number for livestock-associated methicillin-resistant *Staphylococcus aureus* in 62 hospitals in the Netherlands.
