# Supplementary material for: Transmissibility of Livestock-associated Methicillin-Resistant Staphylococcus aureus
Source: Emerg Infect Dis. 2013 Nov;19(11):1797–802. doi: 10.3201/eid1911.121085 (PMC3837675; doi:10.3201/eid1911.121085)
Supplement: Technical Appendix — Statistical methods used to calculate single-admission reproduction number for livestock-associated methicillin-resistant Staphylococcus aureus in 62 hospitals in the Netherlands. [file 12-1085-Techapp-s1.pdf]

Article DOI: http://dx.doi.org/10.3201/eid1911.121085

Transmissibility of Livestock-associated Methicillin-Resistant *Staphylococcus aureus*

Technical Appendix

Statistical Methods

The model of Bootsma et al.(*1*) predicts that the detected size of an outbreak is geometrically distributed. We denote the parameter of the geometric distribution by ξ. This means that the likelihood that a randomly chosen detected outbreak has size *i* (including the index case) is given by $\xi(1-\xi)^{i-1}$.

If there are *N* outbreaks with a total of *M* secondary cases, the likelihood of observing these outbreak sizes is given by $L = \xi^N(1-\xi)^M$. The maximum likelihood estimator, i.e., the value of the parameter ξ which makes the observations most likely, is given by $\xi_{MLE} = N/(N + M)$.

A confidence interval for ξ_{MLE} can be obtained by the profile likelihood method. This confidence interval contains all values for the parameter ξ of the geometric distribution for which the observations are still sufficiently likely. The cutoff values to determine whether the data are still sufficiently likely depends on whether we are calculating 90% confidence intervals, 95% or 99% confidence intervals and is based on the chi-square distribution.

When we know the discharge rate and the rate at which colonization is detected, we can calculate a ratio of these two. With this ratio (*r*) we can translate values for ξ into values of R_A, the per admission reproduction number by using the formula $R_A = (1- \xi)(r + \xi) / \xi$ (see Bootsma et al. for more details).

To check whether the model assumptions, which lead to a geometric distribution of the outbreak sizes, are in agreement with the data, we tested whether the observed outbreak sizes are indeed similar to a geometric distribution by performing the Anderson Darling goodness of fit

test. This test is based on the test statistic $A=\sum_{i=1}^{\infty} p(i)\frac{(\hat{F}(i)-F(i))^2}{F(i)(1-F(i))}$ where $p(i) = \xi_{MLE}(1-\xi_{MLE})^{i-1}$ is the probability density function of the geometric distribution, $F(i)$ is the cumulative density function corresponding to $p(i)$ and $\hat{F}(i)$ is the empirical cumulative density function.

Reference

1. Bootsma MC, Wassenberg MW, Trapman P, Bonten MJ. The nosocomial transmission rate of animal-associated ST398 meticillin-resistant *Staphylococcus aureus*. J R Soc Interface. 2011;8:578–84. PubMed http://dx.doi.org/10.1098/rsif.2010.0349

Technical Appendix Table A. Mathematical parameters of index cases*

Parameter	LA-MRSA, n = 40	Other (non LA)-MRSA,n = 101
Discharge rate (days)	1/13	1/10
Detection rate (days)	1/20	1/20
ξ (95% CI)	0.93 (0.83–0.98)	0.70 (0.62–0.77)
R_A (95% CI)	0.12 (0.03–0.30)	0.52 (0.38–0.69)

*LA-MRSA, livestock-associated methicillin-resistant *Staphylococcus aureus*.

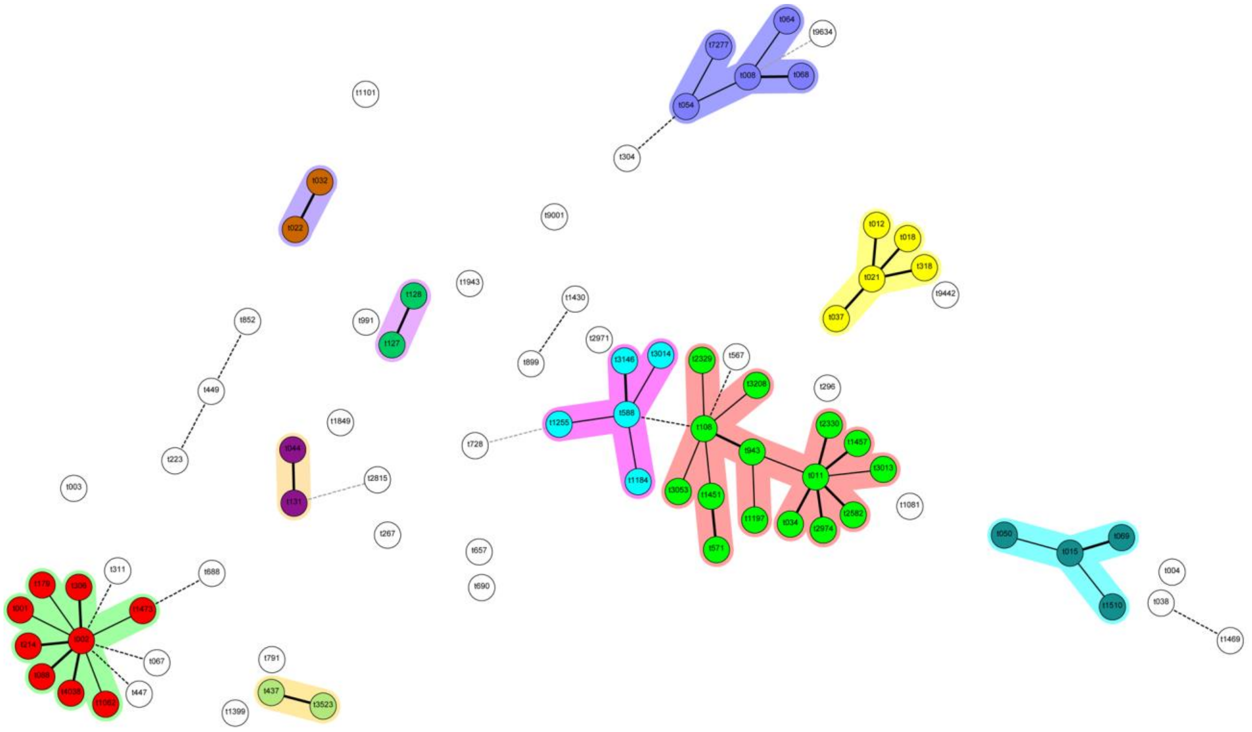

Technical Appendix Figure. *spa*-based minimal spanning tree, including *spa* types of index cases and *spa* types considered livestock associated. *spa* types with the same color are considered to be related.
